# Contagious Diseases

**Published:** 1881-11

**Authors:** 


HALL'S
JOURNAL OF HEALTH
AND
MISCELLANY.

E. H. GIBBS, A. M., M. D. - - EDITOR.

Founded in 1854, and published Monthly without Interruption since that Time.

### CONTAGIOUS DISEASES.

IT was formerly supposed that consumption could only exist as an hereditary disease; that is, that tubercles, which characterize the disease, must have existed in an undeveloped state from birth, and that their presence was a perpetual menace to the life of the individual; and that their non-existence implied immunity from the dreaded disease. But careful investigation proves three things, the knowledge of which is of vast importance to every human being.

*First.* That such care of the health as would be wise under any circumstances is usually sufficient to ward off the disease in spite of hereditary tendencies.

*Second.* That a large proportion of the victims of consumption have not the slighest hereditary tendency in that direction.

*Third.* That consumption is contagious, and quite as likely to be propagated in this way as in any other.

Let me direct the attention to a few familiar facts. Some of my readers may, in looking back twenty years, remember having been acquainted with two families, comprising perhaps eight members in each. One family seemed to be perpetually on the defensive against attacks of disease incident to delicate constitutions, but which attacks were met intelligently and overcome. The other, confident in its inherited vigor and health, never learned prudence. In a great majority of these cases, it will be found that at the end of the score there will be more survivors in the first family than in the second, and moreover that the average condition of health of the first family will be better than that of the second. The increase of twenty years in the average length of human life in this century is due more to a better knowledge of the conditions essential to our well being than to any marked change in the physical condition of the race. I would not wish to be understood as saying that there has been no improvement in this respect; there doubtless has been; but it is certain that a careful observance of the "laws of health" will carry a delicate person farther along the scale of life than the most robust could hope to attain without it.

But let us return to the question of contagion, since there is no subject of more interest and importance than a knowledge of the methods by which diseases are transmitted. Under all circumstances it should be borne in mind that the demands of prudence are inexorable, however sad or cruel or arbitrary they may seem.

A young mother recently lost her only child of diphtheria. The dead child was about to be consigned to the grave, when the mother insisted on kissing it. She thus contracted diphtheria and died within a week afterward. It requires the exercise of cold philosophy to restrain the impulses of a mother's nature; but here it would have saved a precious life.

A few years ago there lived in Boston a sea captain, who was a devoted husband and kind father. He went with his ship to China, and while there contracted leprosy, the most dreadful of diseases, since there is no cure for it. Thus he found himself the victim of a disease more terrible than death, since it cut him off forever from the companionship of all who were dearest to him on earth.

With the heroism worthy of a martyr, he disappeared under circumstances that indicated that he had fallen into the sea and been drowned, and a message to that effect was sent to his family. Instead of drowning, however, he took passage on a vessel bound to the Sandwich Islands. There he entered the hospital for lepers and died four years later.

Think of the courage it required to subdue his longing for home and for kindred during these four dreary years, and if fate has been cruel to you, know that others have drained the cup of misery.

No person should expose his life unnecessarily. There may be occasions when it would be noble to do so and dastardly to refuse; but, ordinarily, there is no excuse for it. No person, whose presence is not required, should remain long at a time in a close room near a person who is ill of any disease whatever. The breath and the emanations from the bodies of sick people are detrimental to the health of all who are much exposed to their influence.

---

LIFE is too short for its possessors to wear long faces.

